# Oxidative Stress and Antioxidant Defense Mechanisms in Response to Starvation and Refeeding in the Intestine of Stellate Sturgeon (*Acipenser stellatus*) Juveniles from Aquaculture

**DOI:** 10.3390/ani11010076

**Published:** 2021-01-04

**Authors:** Iulia Elena Florescu (Gune), Sergiu Emil Georgescu, Andreea Dudu, Mihaela Balaș, Sorina Voicu, Iulia Grecu, Lorena Dediu, Anca Dinischiotu, Marieta Costache

**Affiliations:** 1 Department of Biochemistry and Molecular Biology, Faculty of Biology, University of Bucharest, 050095 Bucharest, Romania; iuliaflorescu_2006@yahoo.com (I.E.F.); tn_andreea@yahoo.com (A.D.); mihaela.radu@bio.unibuc.ro (M.B.); sori.petrache@yahoo.com (S.V.); anca.dinischiotu@bio.unibuc.ro (A.D.); marieta.costache@bio.unibuc.ro (M.C.); 2 Department of Aquaculture, Environmental Sciences and Cadastre, Faculty of Environmental Science and Biotechnology, “Lower Danube” University of Galați, 800201 Galați, Romania; iulia.grecu@ugal.ro (I.G.); lorena.dediu@ugal.ro (L.D.)

**Keywords:** stellate sturgeon, aquaculture production, antioxidant enzymes, oxidative stress, starvation/refeeding regime

## Abstract

**Simple Summary:**

The stellate sturgeon (*Acipenser stellatus*) is a critically endangered species due to multiple causes including human impact, with its valuable roe being used for caviar production. As a consequence, it is intensively raised in aquaculture for both conservation and economical purposes. Food deprivation can occur in both natural and aquaculture environments due to specific conditions that impose food restrictions (such us extreme temperatures, deterioration of water quality, pathological manifestations). The purpose of this study was to investigate the physiological adaptability of stellate sturgeon under two starvation/refeeding regimes. Therefore, the study had two objectives: to assess the effects of an alternative feeding regime on oxidative stress biomarkers and to assess the antioxidant defense mechanisms in juveniles raised in aquaculture. The results showed that the starvation/refeeding regimes induced lipid peroxidation and an enhancement of antioxidant enzymes activities in the intestine of stellate sturgeon juveniles. To conclude, *Acipenser stellatus* proved to possess a potential to adapt to a starvation/refeeding regime, the most suitable being a 7-day starvation period followed by 21 days of refeeding. This regime could be useful to optimize the feeding practice in aquaculture production in order to increase the profitability of fish farming without affecting the stellate sturgeon juveniles.

**Abstract:**

*Acipenser stellatus* is a critically endangered species due to the anthropic influence. It has been intensively captured for decades because of its high economic value, its roe being used in the caviar industry. Therefore, *Acipenser stellatus* is intensively raised in fish farms for both conservation and economical purposes. Aquaculture aims to optimize the feeding regime of juveniles in order to improve its profitability. The purpose of this study was to investigate if *Acipenser stellatus* can adapt to a starvation/refeeding regime by assessing the effects of this regime on oxidative stress biomarkers and antioxidant defense mechanisms in juveniles raised under aquaculture conditions. The juveniles were subjected to two regimes: a 7-day starvation period followed by 21 days of refeeding, respectively a 14-day starvation period followed by 21 days of refeeding. The results showed that both starvation/refeeding regimes induced an enhancement of antioxidant enzymes activities in the intestine of the juveniles. The oxidative damage was counteracted at the protein level. However, lipid peroxidation was significantly induced in the intestine of the juveniles subjected to 14/21-day starvation/refeeding regime. The 7/21-day starvation/refeeding regime proved to be more suitable for *Acipenser stellatus* and therefore, it could be useful to optimize the feeding practice in aquaculture production.

## 1. Introduction

*Acipenser stellatus* (Pallas 1771), also called stellate sturgeon, belongs to an ancient group of fishes that appeared in the Jurassic period. This sturgeon species lives and eats in Black Sea and migrates in the Danube River in order to reproduce [1]. The stellate sturgeon possesses an outstanding scientific value because it remarkably survived two mass extinctions and several Ice Ages, being considered a “living fossil”. Moreover, *A. stellatus* has a high economical value due to its roe that is intensively used in the caviar industry. Unfortunately, because of its great economical value, the stellate sturgeon has been massively captured. Overfishing, alongside the construction of the Iron Gates Dams over the Danube River that impaired the upstream migration and spawning of *A. stellatus*, has led to an alarming decline of this sturgeon population in the Black Sea [2]. As a consequence, *A. stellatus* became extinct in the Upper and Middle Danube and it is considered critically endangered in the Lower Danube River [3,4]. Therefore, stellate sturgeon is intensively raised in fish farms, the individuals bred in aquaculture being used either in restocking programs or for the production of caviar, aiming to discourage poaching and overfishing of wild individuals. 

Because a large part of sturgeon farming is devoted to caviar production, long production cycle technology is applied. In order to keep the activity within sustainability limits, fish farmers aim to optimize the feeding management and, in this pursuit, they are currently trying to introduce food deprivation periods in the feeding practice. This is performed for multiple reasons. First of all, this would increase the profitability of fish farming, given that feeding represents at least 50% of the production cost [5]. This represents the highest cost in an intensive aquaculture system [6]. Secondly, food deprivation is sometimes performed in fish farming in order either to reduce water pollution and to decrease mortality caused by disease outbreaks [7] or to improve preservation of fish stock before marketing and slaughtering [8].

Fishes alternate fasting with feeding periods in their natural existence because of seasonal variations in food availability from natural habitats and due to reproduction and migration habits; therefore, fishes are well adapted to starvation [9,10]. They adopt different strategies in order to survive starvation periods; precisely they reduce the energetic demands either by decreasing the mass of the tissues with high turnover rates or by lowering the metabolic rates [11]. Based on the idea that fishes are adapted to food deprivation in natural environments, several studies have been conducted to assess if a regime based on a starvation period followed by refeeding affects the growth performance and the welfare of the fishes reared in aquaculture conditions. 

Morphometric, hematological, biochemical, metabolic parameters and oxidative stress biomarkers were determined mainly in fish juveniles, but also in adults subjected to different starvation/refeeding regimes. Siberian sturgeon (*Acipenser baerii*) [12], Persian sturgeon (*Acipenser persicus*) [13] and Chinese sturgeon (*Acipenser sinensis*) [14] presented a complete compensatory growth as a response to short-term starvation, while *A. sinensis* [14] and *A. persicus* [13] showed a partial compensation when subjected to long-term starvation periods. The mechanism of compensatory growth in animals has been described as involving the growth hormone, insulin and insulin-like growth factor [15]. However, beluga (*Huso huso*) did not present a complete catch-up growth when subjected to different starvation/refeeding regimes, although the growth rate of the individuals was high [16]. In contrast to the above mentioned species, other fish species, such as channel catfish (*Ictalurus punctatus*) [17] and red porgy (*Pagrus pagrus*) [7] failed to present a compensatory growth response when subjected to different starvation/refeeding regimes. 

Adaptive responses to short or long-term starvation regarding hematological, biochemical or metabolic parameters were found in *A. baerii* [18], Adriatic sturgeon (*Acipenser naccarii*) and rainbow trout (*Oncorhynchus mykiss*) [19], European eel (*Anguilla anguilla*) [20], tench (*Tinca tinca*) [21], common dentex (*Dentex dentex*) [22] and European sea bass (*Dicentrarchus labrax*) [23]. 

Moreover, starvation/refeeding regimes were proved to enhance cell protective mechanisms, such as antioxidant defense mechanisms and heat shock protein (Hsp) expression in *D. labrax* [24]. Even though the reported results differ amongst species, it was mostly observed that fishes do present a metabolic adjustment to minimize the energy expenditure during starvation and an adaptive response to cope with oxidative stress that may be induced by a starvation/refeeding regime. 

In this context, the present study aimed to determine if *A. stellatus* juveniles raised in aquaculture have the ability to adapt to a starvation/refeeding regime by assessing the effects of this alternative type of feeding on oxidative stress biomarkers and antioxidant defense mechanisms in the intestine. The specific activities of the major antioxidant enzymes were analyzed, such as catalase (CAT), superoxide dismutase (SOD), glutathione peroxidase (GPx), glutathione reductase (GR), glutathione S-transferase (GST) and glucose 6-phosphate dehydrogenase (G6PDH). Additionally, the content of reduced glutathione (GSH) was determined alongside the level of several oxidative stress biomarkers: malondialdehyde (MDA), protein thiol groups (PTG), advanced oxidation protein products (AOPP) and protein carbonyl groups (PCG). The present work extends a previous study conducted by our research group on stellate sturgeon liver regarding the adaptability of this species to a starvation/refeeding regime [25]. There is a strong necessity to expand the study on other organs in order to assess the real adaptability of this species to a starvation/refeeding regime. No other similar studies were conducted before on *A. stellatus* individuals, so the results regarding the adaptability of stellate sturgeon individuals to starvation and refeeding are novel and expand the knowledge of stellate sturgeon physiology. Moreover, the oxidative stress biomarkers from the small intestine of sturgeon were not studied before under food deprivation conditions. This study is of great interest for the aquaculture field given that the results represent a starting point in optimizing the feeding regime of *A. stellatus* juveniles from fish farms. A regime based on starvation and refeeding periods could decrease the costs of raising juveniles and eventually increase the profitability of fish farms, stimulating aquaculture practice. In the long run, this could enhance the efforts to conserve *A. stellatus*, and therefore, this study is important from both economical and conservation perspectives. 

As it will be seen further on, the study concluded that several antioxidant defense mechanisms were enhanced by the starvation/refeeding regimes in the intestine of *A. stellatus* juveniles; overall, proteins were spared by the oxidative damage. Lipid peroxidation was induced significantly only in the intestine of the juveniles subjected to a longer starvation period. *A. stellatus* possess a potential to adapt to a starvation/refeeding regime, the 7-day starvation period followed by 21 days of refeeding being better tolerated than the 14-day starvation period followed by 21 days of refeeding. This alternative type of feeding is worth to be further researched and optimized so that it could be eventually applied in stellate sturgeon fish farming. As mentioned above, the results presented in this study must be regarded as a starting point in optimizing the feeding regime of *A. stellatus* juveniles due to several limitations we confronted with. Precisely, the number of individuals available for performing the study was relatively low. We experienced the problem of assessing numerically small groups of individuals due to the fact that *A. stellatus* is a critically endangered species. Moreover, the high costs of raising stellate sturgeon juveniles in fish farms and the difficulty of attaining a successful reproduction of sexually mature individuals made even harder to obtain and study a high number of juveniles. Therefore, for both ethical and economical purposes the number of the juveniles was reduced as much as it was scientifically possible. As a consequence the study presents two issues. Firstly, the treatments were not replicated and the fact that the individuals subjected to a particular treatment were reared together raises the issue of pseudo replication. Secondly, fish in different treatments were sampled on different days and there was no control group for each sampling point. We were obliged to use only one control group due to the limited number of fish. By highlighting these critical aspects we underline the preliminary nature of the data presented in the study.

## 2. Materials and Methods

Animal experimental procedures were in accordance with the Guide for The Use and Care of Laboratory Animals (National Research Council (US) Committee for the Update of the Guide for the Care and Use of Laboratory Animals 2011) and all the efforts were made to minimize animal suffering and reduce the number of specimens used. All animal experiments were approved by the Ethics Committee from “Lower Danube” University of Galați (Approval ID: 200/22.12.2014).

### 2.1. Experimental Design

In the present study, 48 *Acipenser stellatus* individuals of one year age and the mean weight of 331.3 ± 71.8 g were purchased from a sturgeon farm (Tulcea County, Romania). They were randomly distributed in six fiberglass reinforced polyester tanks of 1 m^3^ capacity in a recirculating system (8 individuals per tank). The tanks were maintained at the natural day length in May-June at our latitude. The water flow was 6 m^3^ h^−1^ and the evacuated water was treated with special filters (drum filter, rotating sieve screen, activated sand bed filter, activated carbon filter, denitrification filter, biological filter for wastewater treatment) and sterilized with UV light before admission into the system; the water temperature, pH level and oxygen concentration were maintained at a mean value of 25 °C ± 1, 7.70 ± 0.29, respectively 6.60 ± 0.44 mg L^−1^; the concentrations of ammonium, nitrites and nitrates were monitored during the whole experiment and maintained at a mean value of 0.43 ± 0.12, 0.32 ± 0.15 and 32.20 ± 17 mg L^−1^ respectively. 

The juveniles were acclimatized for two months before the initiation of the experiment. After this period, the juveniles were subjected to the following treatments:

1. The fed control group (Fed C) was subjected to the classical feeding regime applied in aquaculture: juveniles were fed three times per day (at 9:00, 13:00 and 17:00 h) with Troco PreGrower commercial pellets (Table 1), the quantity of food given during a meal being equal to 1% of the total biomass; this regime was applied for the entire period of experiment;
2. A group was subjected to a 7-day starvation period and sampled (7 S group);
3. A group was subjected to a 14-day starvation period and sampled (14 S group);
4. A group was starved for 7 days and refed for 21 days (7 S-R group)—this regime being the 7/21-day starvation/refeeding regime;
5. A group was starved for 14 days and refed for 21 days (14 S-R group) —this regime being the 14/21-day starvation/refeeding regime;
6. A group was starved for the entire period of experiment (S C group—starved control group).

The refeeding process of the 7 S-R group and 14 S-R group was performed in the same manner as in the Fed C group. The entire experiment lasted 35 days. Fed C and S C groups were sampled at the end of the experiment. 

The fish were anesthetized in a bath with 0.3 mL L^−1^ 2-phenoxyethanol before sampling. Afterwards, they were sacrificed by cutting the gill arch and the small intestine of each individual was collected and put into sterile tubes on ice and kept at −80 °C for biochemical analyses. All analyses were performed in triplicates.

### 2.2. Biochemical Analyses

#### 2.2.1. Preparation of the Total Protein Extracts

Fragments of 0.1 g from intestine tissue were mixed with 1 mL of ice-cold TRIS-EDTA buffer (0.1 M TRIS-HCl buffer containing 5 mM EDTA, pH 7.4) and homogenized (seven times, 30 s each) on ice using a UP 50H sonicator (Hielscher). After 30 min centrifugation of tissue homogenates at 10,000 rpm, 4 °C, the resulting supernatants were collected and preserved at −80 °C for further analyses. The total protein concentration was measured according to the method described by Lowry [26] using 0.01 g mL^−1^ bovine serum albumin (BSA) as standard. 

#### 2.2.2. Assessment of Antioxidant Enzymatic Activities

The activities of antioxidant enzymes were assessed at 25 °C using a Specord 200 Plus spectrophotometer or a Flex Station 3 Multireader and the reagents were purchased from Sigma Aldrich or Merck.

Catalase (CAT; EC 1.11.1.6) activity was assessed according to the method described by Beers and Sizer (1952) by monitoring the decrease of hydrogen peroxide (H_2_O_2_) concentration at 240 nm [27]. One unit of enzyme (U) decomposes one µmole H_2_O_2_ in one minute at 25 °C and pH 7.

Superoxide dismutase (SOD; EC 1.15.1.1) activity was assessed according to the method described by Paoletti et al. (1986) by monitoring the inhibition of NADH oxidation for 21 min at 340 nm [28]. NADH oxidation was mediated by superoxide anion that was generated from molecular oxygen in a chemical reaction involving β-mercaptoethanol, triethanolamine, diethanolamine, EDTA and manganese chloride. One unit of SOD was described as the amount of enzyme required to inhibit the NADH oxidation rate by 50% compared to the control. 

Glutathione peroxidase (GPx; EC 1.11.1.9) activity was assessed according to the method described by Beutler (1984) by monitoring the reduction of tert-butyl-hydroperoxide and the oxidation of reduced glutathione (GSH) to oxidized glutathione (GSSG) [29]. Further on, the resulting GSSG was reduced to GSH by glutathione reductase and conversion of NADPH to NADP^+^ was measured at 340 nm. One unit of GPx was defined as the amount of enzyme required to consume one µmole NADPH in one minute at 25 °C.

Glutathione reductase (GR; EC 1.6.4.2) activity was assessed according to the method described by Goldberg and Spooner (1986) by monitoring the decrease of NADPH concentration at 340 nm following the conversion of GSSG to GSH [30]. One unit of GR was defined as the amount of enzyme required to consume one µmole NADPH in one minute at 25 °C.

Glutathione S-transferase (GST; EC 2.5.1.18) activity was assessed according to the method described by Habig et al. (1974) by monitoring the conjugation of GSH with 1-chloro-2,4-dinitrobenzene (CDNB) substrate at 340 nm [31]. One unit of GST was defined as the amount of enzyme necessary to form one µmole of GS-CDNB product in one minute at 25 °C.

Glucose 6-phosphate dehydrogenase (G6PDH; EC 1.1.1.49) activity was measured by monitoring the increase of NADPH concentration at 340 nm. The enzyme catalyzes the oxidation of glucose 6-phosphate by reducing NADP^+^ [32]. One unit of G6PDH was defined as the amount of enzyme required to produce one µmole NADPH in one minute at 25 °C. 

The specific activities of all enzymes were calculated as U mg^−1^ protein using the specific molar extinction coefficients (ε_H2O2_ = 43.6 × 10^3^ M^−1^ cm^−1^, ε_NADPH_ = 6.22 × 10^3^ M^−1^ cm^−1^, ε_CDNB_ = 9.6 × 10^3^ M^−1^ cm^−1^).

#### 2.2.3. Reduced Glutathione Assay

The content of GSH was evaluated using Glutathione Assay Kit from Sigma-Aldrich (Saint Louis, MO, USA) according to the manufacturer’s instructions. Briefly, tissue lysates were firstly deproteinized with one volume of 5% sulfosalicylic acid followed by centrifugation for 10 min at 10,000 rpm, 4 °C. Afterwards, a volume of supernatant was mixed with 1.5 mg mL^−1^ 5, 5′-dithio-bis-2-nitrobenzoic acid (DTNB) in potassium phosphate buffer solution pH 7 and the amount of the resulted TNB products was measured at 405 nm. A calibration curve with standard GSH solution of various concentrations in the range of 3.1–400 µM was used in order to determine the GSH concentration in the samples. 

#### 2.2.4. Malondialdehyde Assay

Malondialdehyde (MDA) is a lipid peroxidation end product that was determined using 2-thiobarbituric acid (TBA) according to a classical method [33]. A volume of 50 µL of sample was mixed with 175 µL of 0.1 N HCl and incubated for 20 min at room temperature. After adding 225 µL of 0.025 M TBA, the mixture was incubated for another 65 min at 37 °C. The fluorescence of MDA-TBA adducts was recorded at 520 nm excitation and 549 nm emission using Flex Station 3 multireader. A calibration curve with standard MDA (1,1,3,3-tetraethoxypropane) ranging between 0.025 and 0.5 µM was used to calculate the concentration of MDA in the samples. 

#### 2.2.5. Assessment of Protein Oxidation Biomarkers

Protein thiol groups (PTG) concentration was determined with 4,4′-dithiodipyridine (DTDP) [34]. Protein precipitation of the samples was performed with 20% trichloroacetic acid (TCA) on ice. After centrifugation, the protein containing pellet was solubilized in 1M NaOH and incubated with 4 mM DTDP for 5 min at room temperature in the dark. The absorbance of the DTDP conjugated thiol groups was measured at 324 nm. A calibration curve with standard N-acetyl-cysteine ranging between 0.5 and 40 µM was used to calculate the concentration of PTG in the samples. The concentration of the PTG was divided by the total protein concentration of the sample and it was expressed as μM mg^−1^ protein. 

Protein carbonyl groups (PCG) concentration was determined by measuring the hydrazones resulted in the reaction of 2,4-dinitrophenylhydrazine (DNPH) with protein carbonyls [35]. Protein extracts were incubated with 10 mM DNPH for one hour at room temperature in the dark. After protein precipitation with ice-cold 20% trichloroacetic acid (TCA), the samples were centrifuged; the pellet was washed two times with ethanol: ethyl acetate mixture and the absorbance of the carbonyl-DNPH products were measured at 370 nm. The concentration of PCG was calculated using ε_DNPH_ = 22,000 M^−1^ cm^−1^ as molar extinction coefficient. The concentration of PCG was divided by the total protein concentration of the sample and it was expressed in nmole mg^−1^ protein.

Advanced oxidation protein products (AOPP) were measured spectrophotometrically [36]. Samples were incubated with 1.16 M potassium iodide (KI) for 5 min at room temperature. Glacial acetic acid was added to the mixture and the optical density was recorded at 340 nm. A calibration curve with chloramine T of various concentrations (5–100 µM) was used to quantify the AOPP content in the samples. The concentration of AOPP was divided by the total protein concentration of the sample and it was expressed as μM mg^−1^ protein. 

### 2.3. Statistical Analysis

Technical replicates were averaged before the statistical analysis. The data are illustrated as average value of the group (n = 8) ± standard error of the mean (SEM). All data were statistically analyzed using one way-ANOVA method performed with Graph Pad Prism 3.03 software (GraphPad Software, La Jolla, CA, USA). Post-hoc comparisons between all groups were run using Bonferroni test. If *p* value was lower than 0.05 then the difference between the groups was considered statistically significant (* *p* < 0.05; ** *p* < 0.01; *** *p* < 0.001). The statistical significance was presented for all groups as contrast to the Fed C group and it was also presented between 7 S and 14 S groups and between 7 S-R and 14 S-R groups.

## 3. Results

### 3.1. Antioxidant Defense Mechanisms

**Figure 1:**
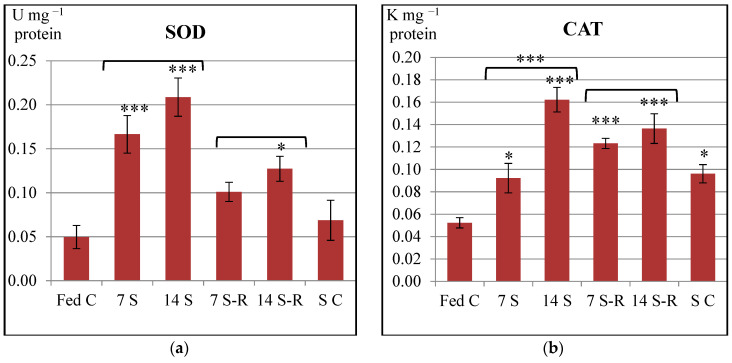
Specific activities of antioxidant enzymes in the intestine of *Acipenser stellatus* juveniles subjected to different starvation/refeeding regimes. (**a**) Superoxide dismutase (SOD) specific activity; (**b**) catalase (CAT) specific activity. The data are illustrated as average value of the groups (n = 8) ± standard error of the mean (SEM). All data were statistically analyzed using one way—ANOVA. Statistical significance: * *p* < 0.05; *** *p* < 0.001; the statistical significance of the changes is related to the fed control level and it is also presented between 7 S and 14 S groups and between 7 S-R and 14 S-R groups. Fed C—fed control group; 7 S—group starved for 7 days; 14 S—group starved for 14 days; 7 S-R—group starved for 7 days and refed 21 days; 14 S-R—group starved for 14 days and refed 21 days; S C—starved control.

SOD and CAT specific activities were enhanced in the intestine of stellate sturgeon juveniles after both 7-day and 14-day starvation periods in comparison to the activities observed after constant feeding (Figure 1). Enhanced activities of both antioxidant enzymes observed during starvation might reflect an increase in the concentration of enzymatic substrates (O^2−^ and H_2_O_2_), which is equivalent to an overproduction of reactive oxygen species (ROS). However, SOD and CAT specific activities were more increased after 14-day starvation period than after 7-day starvation one, the increase of CAT being statistically significant (*p* < 0.001) (Figure 1b). This result could suggest that 14-day starvation period induced oxidative stress in the intestine of stellate sturgeon to a greater extent than 7-day starvation period did.

SOD specific activity decreased and tended to return to the level registered for fed control group when refeeding was performed. Precisely, refeeding after a 7-day starvation period diminished the activity of SOD to a level that was insignificantly higher than the fed control one (*p* > 0.05), while, refeeding after a 14-day starvation period diminished the activity of SOD to a level that was significantly higher than the fed control level (*p* < 0.05) (Figure 1a).

CAT specific activity was strongly higher in the intestine of the juveniles subjected to both starvation/refeeding regimes when compared to fed control, the difference between these experimental groups being highly statistically significant (*p* < 0.001). CAT specific activity increased in the intestine of 7 S-R group in comparison to 7 S group. In contrast, it was diminished in 14 S-R group in comparison to 14 S group. However, the level of CAT activity observed for the 7 S-R group was slightly lower than that observed for the 14 S-R group (Figure 1b).

Considering all the results, the 7/21-day starvation/refeeding regime induced oxidative stress to a lesser extent than 14/21-day starvation/refeeding regime in the intestine of stellate sturgeon juveniles.

The level of SOD specific activity observed for the starved control was similar to the one observed for the fed control (*p* > 0.05), in contrast to the level of CAT specific activity that was significantly higher than the fed control level (*p* < 0.05) (Figure 1).

**Figure 2:**
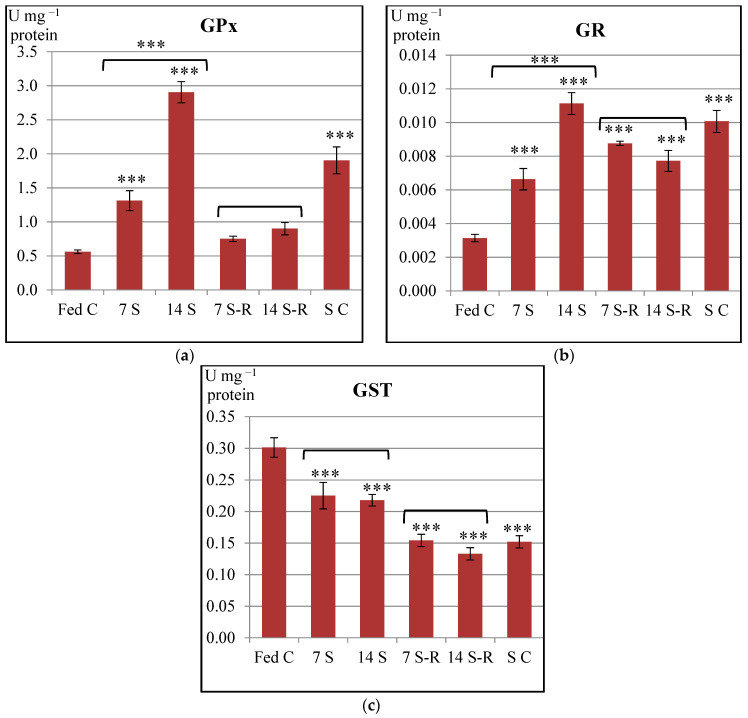
Specific activities of antioxidant enzymes in the intestine of *Acipenser stellatus* juveniles subjected to different starvation/refeeding regimes. (**a**) Glutathione peroxidase (GPx) specific activity; (**b**) glutathione reductase (GR) specific activity; (**c**) glutathione S-transferase specific activity. The data are illustrated as average value of the groups (n = 8) ± standard error of the mean (SEM). All data were statistically analyzed using one way—ANOVA. Statistical significance: *** *p* < 0.001; the statistical significance of the changes is related to the fed control level and it is also presented between 7 S and 14 S groups and between 7 S-R and 14 S-R groups. Fed C—fed control group; 7 S—group starved for 7 days; 14 S—group starved for 14 days; 7 S-R—group starved for 7 days and refed 21 days; 14 S-R—group starved for 14 days and refed 21 days; S C—starved control.

**Figure 3:**
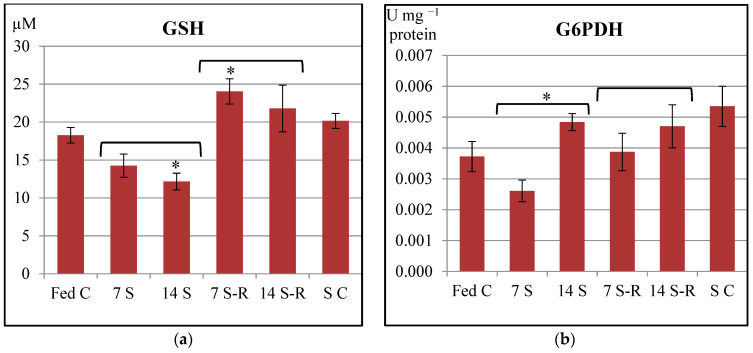
The level of other biomarkers involved in antioxidant defense mechanisms in the intestine of *Acipenser stellatus* juveniles subjected to different starvation/refeeding regimes. (**a**) The level of GSH; (**b**) glucose 6-phosphate dehydrogenase (G6PDH) specific activity. The data are illustrated as average value of the groups (n = 8) ± standard error of the mean (SEM). All data were statistically analyzed using one way—ANOVA. Statistical significance: * *p* < 0.05; the statistical significance of the changes is related to the fed control level and it is also presented between 7 S and 14 S groups and between 7 S-R and 14 S-R groups. Fed C—fed control group; 7 S—group starved for 7 days; 14 S—group starved for 14 days; 7 S-R—group starved for 7 days and refed 21 days; 14 S-R—group starved for 14 days and refed 21 days; S C—starved control.

GPx and GR specific activities increased in a time dependent manner in the intestine of the starved juveniles when compared to the constantly fed juveniles (Figure 2a,b). Thus, the 14-day starved juveniles presented a significantly higher activity of GPx-GR enzymatic system when compared to the 7-day starved juveniles, suggesting that the 14-day starvation induced peroxides to a greater extent than the 7-day starvation did. Even though, GPx activity was higher in the 7 S group compared to fed control, GSH level did not differ significantly between these two groups (Figure 3a). In contrast, the 14 S group presented an increase of GPx activity and a decrease of GSH level, in comparison to fed control level. Therefore, the 14-day starvation period induced an enhancement of GSH utilization by GPx in comparison to 7-day starvation period. 

GPx specific activity decreased when refeeding was performed and even returned to the fed control level in the intestine of the juveniles subjected to both starvation/refeeding regimes (Figure 2a). These results might indicate that the removal of peroxides was performed by GPx. However, GR activity remained significantly higher in both refed groups when compared to fed control group, indicating the necessity to recycle oxidized glutathione, even though the GPx and GST activities were diminished (Figure 2b). These results suggest that both starvation/refeeding regimes enhanced the recycling rate of oxidized glutathione. These observations are confirmed by the level of GSH (Figure 3a). 14 S-R group presented a GSH level that was similar to the one observed for the fed control (*p* > 0.05), while 7 S-R group presented a higher amount of GSH than the fed control (*p* < 0.05). Therefore, 14/21-day starvation/refeeding regime induced a greater mobilization and utilization of GSH by GPx than 7/21-day starvation/refeeding regime did. 

Starved control presented a significantly increased activity of both GPx and GR enzymes in comparison to fed control (*p* < 0.001), while GSH level was similar to the one observed for the fed control (*p* > 0.05).

Interestingly, starvation induced a significant decrease of GST specific activity in comparison to constant feeding, while subsequent refeeding induced an even greater decrease of GST activity in the intestine of *A. stellatus* juveniles (Figure 2c). No visible or statistically significant differences were observed between 7 S and 14 S groups or between 7 S-R and 14 S-R groups. Therefore, both starvation/refeeding regimes diminished the level of electrophilic compounds.

There were no statistically significant differences in the G6PDH specific activity between starved, refed and constantly fed juveniles (Figure 3b). Therefore, both starvation/refeeding regimes did not influence the pentose phosphate shunt in a significant manner in the intestine of *A. stellatus* juveniles. However, there was a significant increase of G6PDH activity in the 14 S group in comparison to 7 S group, suggesting that pentose phosphate shunt is influenced by the duration of starvation. 

### 3.2. Oxidative Stress Biomarkers

**Figure 4:**
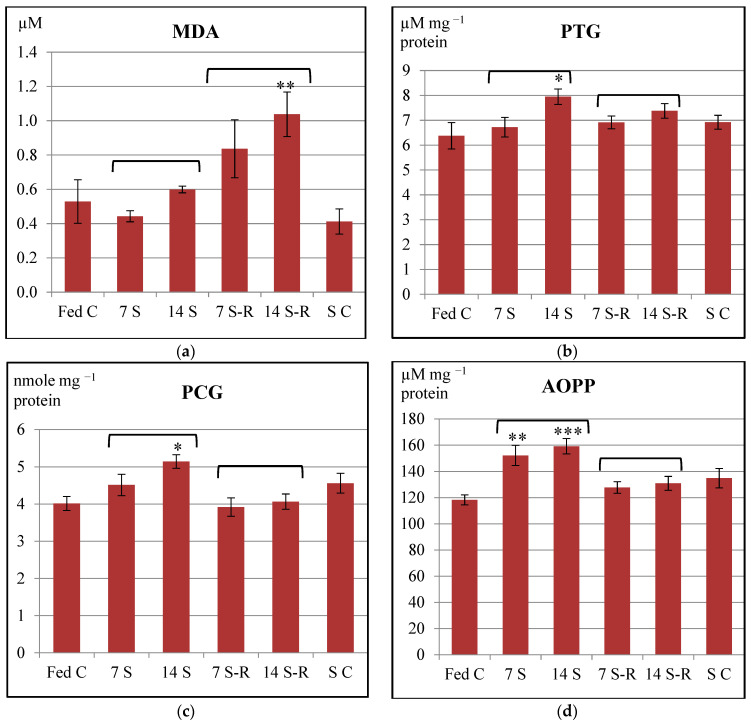
The level of oxidative stress biomarkers in the intestine of *Acipenser stellatus* juveniles subjected to different starvation/refeeding regimes. Lipid peroxidation biomarkers: (**a**) level of malondialdehyde (MDA). Protein oxidation biomarkers: (**b**) concentration of protein thiol groups (PTG). (**c**) The concentration of protein carbonyl groups (PCG). (**d**) The concentration of advanced oxidation protein products (AOPP). The data are illustrated as average value of the groups (n = 8) ± standard error of the mean (SEM). All data were statistically analyzed using one way—ANOVA. Statistical significance: * *p* < 0.05; ** *p* < 0.01; *** *p* < 0.001; the statistical significance of the changes is related to the fed control level and it is also presented between 7 S and 14 S groups and between 7 S-R and 14 S-R groups. Fed C—fed control group; 7 S—group starved for 7 days; 14 S—group starved for 14 days; 7 S-R—group starved for 7 days and refed 21 days; 14 S-R—group starved for 14 days and refed 21 days; S C—starved control.

MDA level slightly decreased during 7-day starvation period and increased during subsequent refeeding in comparison to fed control level, both differences being statistically insignificant (*p* > 0.05) (Figure 4a). In contrast, MDA level slightly increased during 14-day starvation period and continued to increase even greater becoming statistically significant in comparison to fed control level (*p* < 0.01). These results could suggest that both starvation/refeeding regimes induced oxidative stress that was not effectively counteracted by the enhancement of antioxidant defense mechanisms. Therefore, some ROS escaped the scavenging enzymatic systems and induced peroxidation of lipids from the intestine of the *A. stellatus* juveniles. Once more, the results showed that 14/21-day starvation/refeeding regime induced oxidative stress to a higher extent than 7/21-day starvation/refeeding one did.

The content of PTG remained similar amongst 7 S, 7 S-R and Fed C groups (*p* > 0.05), suggesting that the redox status of proteins was not affected by the 7/21-day starvation/refeeding regime. In contrast, the content of PTG significantly raised in 14 S group compared to Fed C group (*p* < 0.05) (Figure 4b). The 14 S group presented not only the highest content of protein thiols, but also the lowest GSH level amongst all analyzed groups (Figure 3a). These results could indicate that the reduction of oxidized protein was mediated by GSH oxidation in the intestine of 14-day starved juveniles. When refeeding was performed, the protein thiol content returned to a level similar to the one observed for the fed control (*p* > 0.05). Therefore, the 14/21-day starvation/refeeding regime induced a mobilization of GSH stocks so that the redox balance of the proteins could be restored.

The level of PCG increased insignificantly after 7-day starvation period, and eventually returned to fed control level after subsequent refeeding (Figure 4c). Therefore, ROS mediated carbonylation of proteins was minimized in the intestine of the stellate sturgeon juveniles subjected to 7/21-day starvation/refeeding regime. In contrast, the level of PCG significantly increased after 14-day starvation period in comparison to fed control level (*p* < 0.05) and decreased after subsequent refeeding. The PCG level of 14 S-R group was similar to fed control level, the difference between the groups being statistically insignificant. Therefore, ROS mediated carbonylation of protein was induced to a certain extent in the intestine of the stellate sturgeon juveniles subjected to 14-day starvation, but was counteracted after subsequent refeeding. 

The AOPP level gradually increased during starvation in comparison to constant feeding. Both 7 S and 14 S groups had a statistically significant higher level of AOPP than Fed C group (Figure 4d). After subsequent refeeding the AOPP level decreased; however, both 7 S-R and 14 S-R groups presented a slightly and insignificantly higher level than the Fed C level. Therefore, protein oxidation was induced to a small degree in the intestine of *A. stellatus* juveniles subjected to both starvation/refeeding regimes.

Overall, analyzing the protein oxidation biomarkers, it could be said that no major oxidative damage of proteins was induced by the starvation/refeeding regimes in the intestine of stellate sturgeon juveniles.

## 4. Discussion

Free radicals, such as reactive oxygen species (ROS), are generated in physiological conditions, being by-products of the aerobic cellular metabolism [37,38]. ROS are highly reactive molecules that contain an impaired electron, and therefore they have a major oxidant effect [37,38]. There are several enzymatic and non-enzymatic antioxidants that neutralize ROS, either by direct reduction of ROS or by repairing the ROS-mediated damage. An imbalance between the production of ROS and the antioxidant defense mechanisms leads to oxidative stress [37,38,39]. This consists of high level of ROS that was not counteracted by the antioxidant mechanisms and in turn, causes oxidative damage to proteins, lipids and other biomolecules. CAT and SOD are two major antioxidant enzymes that represent a primary defense mechanism against ROS. SOD catalyzes the dismutation of O^2−^ into H_2_O_2_, which is further decomposed in the reaction catalyzed by CAT into water and oxygen, preventing formation of the hydroxyl ion. The activities of these enzymes are considered biomarkers for oxidative stress [37,38,39,40].

In our study, SOD and CAT specific activities were increased in the intestine of stellate sturgeon juveniles subjected to both 7-day and 14-day starvation periods in comparison to constantly fed juveniles. Therefore, starvation induced an overproduction of ROS, precisely O^2−^ and H_2_O_2_ that triggered an enhancement of both SOD and CAT activities in the intestine. Similar to our results, both SOD and CAT activities increased in the intestine of one year old *Dicentrarchus labrax* (European sea bass) starved for three weeks [24], in the liver of sexually immature *Dentex dentex* (common dentex) starved for five weeks [41] as well as in the liver and gills of *Salmo trutta* (brown trout) starved for 49 days [42]. Additionally, CAT activity was reported to increase in the liver of *Gadus morhua* (Atlantic cod) after a 12-week starvation period [43]. 

On one hand, SOD activity increased, while CAT activity decreased in the liver of sexually immature *Sparus aurata* (gilthead sea bream) subjected to partial or total food deprivation for 46 days [44]. On the other hand, SOD activity was not affected by starvation, while CAT activity increased in the blood of *Mesopotamichthys sharpeyi* fingerlings starved for 16 days [45]. In contrast to our results, both SOD and CAT activities decreased in the liver of one year old *Acipenser naccarii* (Adriatic sturgeon) and *Oncorhynchus mykiss* (rainbow trout) starved for 72 days [46].

Further on, our study demonstrated a tendency of returning the enzymatic activity of SOD to a control level during refeeding. Eventually, SOD activity reached the control level during 7/21-day starvation/refeeding regime. However, the enzymatic activities of both SOD and CAT remained still higher than the control level during 14/21-day starvation/refeeding regime. Therefore, it might be considered that the production level of ROS observed after refeeding could have been diminished in comparison to the level induced by starvation, but remained higher than the physiological ROS level produced by constant feeding.

In accordance to our results, SOD and CAT activities remained still higher than the control level in the intestine of *Dicentrarchus labrax* refed for two weeks [24] and in the liver and gills of *Salmo trutta* refed for 21 days [42].

In contrast to our results, SOD and CAT activities were recovered and returned to control values after refeeding in the liver of *Dentex dentex* [41], in the blood of *Mesopotamichthys sharpeyi* fingerlings [45] and in the liver of *Sparus aurata* [44]. Additionally, SOD activity was recovered after two months of refeeding in the liver of *Acipenser naccarii* and *Oncorhynchus mykiss*, while CAT activity was recovered in the latter [46]. 

GPx catalyzes peroxides removal by oxidizing GSH, while GR recycles the resulting oxidized glutathione (GSSG) by reducing the disulfide bond, thus producing two molecules of GSH. GPx substrates are both lipid peroxides and H_2_O_2_, the latter being also removed by CAT [38,39,47].

GSH represents an important non-enzymatic cellular antioxidant. It is a tripeptide involved not only in the GPx mediated removal of lipid peroxides and H_2_O_2_, but also in the reduction of oxidized thiol groups of proteins [47]. It is also used by GST to conjugate electrophilic compounds in order to render less chemically active compounds and to ensure their clearance from the organism [48].

In our study, GPx and GR specific activities increased in a time dependent manner in the intestine of starved *Acipenser stellatus* juveniles, suggesting that starvation induced peroxides. The increase of GPx activity resulted in a slightly decreased MDA level after 7-day starvation and only a statistically insignificant increase of MDA level was induced after 14-day starvation when compared to constant feeding. Therefore, GPx might have been involved solely in the detoxification of MDA, whereas H_2_O_2_, the other substrate of GPx might have been removed entirely in the reaction catalyzed by CAT, due to the fact that CAT has a higher value of K_M_ constant for H_2_O_2_ compared to GPx [49]. Therefore, the GPx mediated removal of peroxides and the recycling of glutathione catalyzed by GR were two enzymatic defense mechanisms triggered in the intestine of stellate sturgeon in response to the oxidative stress induced by starvation. Similar to our results, both GPx and GR activities were increased by starvation in the liver of *Salmo trutta* [42] and of *Sparus aurata* [44]. Additionally, GPx activity was enhanced after starvation in the liver of *Gadus morhua* [43] and the blood of *Mesopotamichthys sharpeyi* fingerlings [45]. Furthermore, it was reported an increase of GPx activity and a decrease of GR one in the liver of starved *Dentex dentex* [41]. In contrast to our results, GPx was not affected at all by starvation in the intestine of *Dicentrarchus labrax* [24] and both GPx and GR activities were decreased in the liver of *Acipenser naccarii* and *Oncorhynchus mykiss* [46].

In our study, GPx activity returned to control level after refeeding, suggesting that a reduction of peroxides took place during refeeding. Interestingly, GR activity remained higher after refeeding, even though GPx activity was diminished. This could be explained in the perspective that GR was involved in GSH recycling, which in turn acted on its own as a reducing agent. Precisely, GSH was involved in the reduction of the oxidized protein thiol groups. As a consequence, the redox balance of proteins was effectively maintained. This observation is demonstrated by the content of protein thiol groups. The level of GSH was decreased, while PTG was increased at the end of 14-day starvation period compared to constant feeding. And eventually, both GSH, and PTG levels were similar to control levels after subsequent refeeding.

In agreement to our results, GPx activity was recovered and GR continued to be increased after refeeding in the gills of *Salmo trutta* [42]. In addition, GPx activity returned to control level during refeeding in the liver of *Dentex dentex* [41] and the blood of *Mesopotamichthys sharpeyi* fingerlings [45], and GR activity was increased after refeeding in the liver of *Sparus aurata* [44].

In contrast to our results, GPx activity was either decreased in the liver of refed *Sparus aurata* [44] or increased in the intestine of refed *Dicentrarchus labrax* [24] and in the liver of refed *Salmo trutta* [42]. Additionally, GR activity returned to control value in the liver of refed *Dentex dentex* [41].

Further on, it was observed that the high activity of GPx observed during 7-day starvation did not affect the GSH level which remained similar to the control level. It seems that the increase of GR activity observed during 7-day starvation was sufficient to reduce the oxidized glutathione produced by GPx and to supply GSH. Refeeding after 7-day starvation maintained a high activity of GR despite the fact that the activity of GPx returned to control level. This contributed to the accumulation of GSH in the intestine of refed juveniles. Therefore, the 7-day starvation did not affect the GSH-GSSG balance and the subsequent refeeding increased the level of GSH.

The 14-day starvation induced a major enhancement of both GPx and GR activities and a strong diminish of GSH level. Hence, GSH utilization by GPx was strongly enhanced and GR did not manage to maintain a balance between GSH production and its utilization. However, refeeding after 14-day starvation maintained a high activity of GR despite the fact that the activity of GPx returned to control level. This allowed GSH level to reach the control level.

Therefore, 14/21-day starvation/refeeding regime induced a greater mobilization of GSH stock in the intestine of *A.stellatus* juveniles than 7/21-day starvation/refeeding one.

GST activity was severely reduced by starvation, while subsequent refeeding induced an even greater decrease in the intestine of *Acipenser stellatus* juveniles. It was reported that starvation usually enhances the innate enzymatic capacity of detoxification by up-regulating the expression of genes that are related to antioxidant mechanisms, especially GST [50]. In starved individuals, GPx activity was highly increased compared to constantly fed ones and GSH reserves might have been used with priority as cofactor for GPx than for GST enzyme during starvation. Therefore, it might have been thought that starvation negatively impacted the enzymatic capacity of cellular detoxification mediated by GST. However, even though GPx activity decreased and returned to fed control level after refeeding, GSH content increased instead of being used by GST. So, despite GSH availability, GST activity decreased even more after refeeding compared to fed control. Therefore, we concluded that the diminished GST activity observed during starvation/refeeding regimes did not reflect an incapacity of cellular enzymatic detoxification, but rather a diminished level of electrophilic compounds and that GST had a minor role in the cellular detoxification, whereas GPx was involved in MDA detoxification. 

Similar to our results, GST activity decreased after a 49-day starvation period in the liver of *Salmo trutta*, however, in contrast to our results the activity was recovered after refeeding [42]. In contrast to our results, GST activity raised in the liver of starved *Gadus morhua* [43]. Another study reported that GST activity initially increased after a 7-day starvation period and decreased afterwards at the end of a 28-day starvation period in the liver of *Sparus aurata*. Finally, after refeeding GST activity increased and reached a higher level than control values [44]. 

G6PDH is involved in pentose-phosphate pathway, an alternative metabolic way of glucose oxidation that results in synthesis of NADPH, which is further used by GR to recycle GSSG into GSH when oxidative stress is induced. Therefore, pentose-phosphate shunt is proposed to be the major NADPH source required for the antioxidant defense mechanisms [51]. In our study, G6PDH specific activity was not affected by either starvation or refeeding in the intestine of *A. stellatus*. The activity of G6PDH was increased after the 14-day starvation period in comparison to 7-day starvation suggesting that the pentose-phosphate shunt was intensified during the 14-day starvation, probably due to induction of gluconeogenesis. Therefore, it is likely that the production of glucose through gluconeogenesis maintained an active pentose-phosphate shunt and thus, G6PDH activity might have been unaffected by glucose deprivation even during 14/21-day starvation/refeeding regime. NADPH, which is produced during pentose-phosphate shunt, was most probably used by GR in the reaction of GSH recycling. This is reflected by the significant enhancement of GR activity during 14/21-day starvation/refeeding regime. 

In contrast to our results, G6PDH activity was decreased after starvation and recovered after refeeding in the liver of *Dentex dentex* [41] and the liver and gills of *Salmo trutta* [42].

To conclude, the 7/21-day starvation/refeeding regime did not influence pentose-phosphate shunt in a significant manner, while the 14/21-day starvation/refeeding regime probably induced gluconeogenesis, which sustained this metabolic pathway. Hence, the intestine of stellate sturgeon was able to metabolically adapt to both starvation/refeeding regimes, but the 7/21-day starvation/refeeding regime was better tolerated.

MDA is an iconic biomarker of lipid peroxidation that is produced after ROS mediated oxidation of the polyunsaturated fatty acids (PUFA). The long chains of PUFA are prone to sequential oxidations that could lead to cleavage and render small end products, such as MDA [37,48]. The content of MDA suggests that both starvation/refeeding regimes induced oxidative stress that led to lipid peroxidation. Even though starvation did not induce a great amount of MDA level compared to constant feeding, refeeding induced a higher MDA level than constant feeding. Therefore, the lipid peroxidation was provoked by refeeding. Interestingly, SOD and CAT enzymes presented a highly increased level of activity during refeeding compared to that observed during constant feeding. High levels of SOD and CAT activities should have been prevented the formation of hydroxyl ion which is responsible for lipid peroxidation, and therefore, the lipid peroxidation level should have been maintained to a low value.

Even though the antioxidant defense mechanisms were enhanced, some ROS managed to escape the scavenging enzymatic systems and induced peroxidation of lipids from the intestine of *Acipenser stellatus* juveniles. The metabolic pathways leading to ATP generation such as the mitochondrial electron transport chain might have been diminished during starvation as an adaptive response to the lack of food. The ATP generation might have been enhanced by refeeding in order to compensate for the previous reduction; therefore, a higher amount of ROS might have been induced during refeeding, leading to lipid peroxidation. The intestine of stellate sturgeon juveniles seems to be more prone to lipid peroxidation mediated by ROS that were induced during starvation/refeeding regimes than the liver subjected to the same regimes. The MDA level initially decreased after starvation periods and recovered after refeeding periods in the liver of stellate sturgeon [25]. 

In agreement to our results observed for the intestine of stellate sturgeon, the MDA level was increased during starvation and remained increased after refeeding in the liver and blood of *Acipenser naccarii* and *Oncorhynchus mykiss* [46] and the liver and gills of *Salmo trutta* [42]. In contrast to our results, even though starvation induced a high level of MDA, the level was recovered and returned to control values after refeeding in the liver of *Dentex dentex* [41], the blood of *Mesopotamichthys sharpeyi* [45] and the liver of *Sparus aurata* [44].

The oxidation of protein thiol groups represents a reversible protein modification; the disulfide bonds of oxidized protein can be reduced by GSH compound, and therefore the redox status of the proteins can be restored [52]. PCG and AOPP represent hallmarks of the oxidative stress and are irreversible changes in the proteins [37,53]. PCG consists of aldehydes and ketones that are produced during interaction between the hydroxyl ions and amino acids, such as lysine, arginine, proline and threonine [54]. AOPP are considered to be cross-linked products of proteins that contain dityrosine [53]. Additionally, they could be small products resulting from cleavage of a long polypeptidic chain due to protein oxidation mediated by the hypochlorous acid [52,55]. In our study, the oxidation of protein was induced during starvation periods, as reflected by both AOPP and PCG levels. However, refeeding reduced the carbonylation level that returned to the control one, and decreased the AOPP level that was slightly higher than control level. The oxidized thiol groups of proteins were reduced by GSH during starvation/refeeding regimes, and therefore the redox balance of protein was sustained by the antioxidant defense mechanisms. 

The present work was conducted on the intestine of stellate sturgeon juveniles and therefore, it is hard to properly interpret the results of our study in the perspective of previous studies because most of them were conducted on other organs, mainly liver and blood. As it can be seen, studies reported various results regarding the effects of starvation/refeeding regimes on oxidative stress biomarkers in fish because the species and the age of the individuals, the periods of starvation and refeeding and the methods used are highly different.

Overall, our study proved that starvation/refeeding regimes enhanced the major antioxidant enzymatic systems in the intestine of stellate sturgeon juveniles raised in aquaculture, precisely the 7/21-day starvation/refeeding regime enhanced CAT and GR activities, while the 14/21-day starvation/refeeding regime enhanced SOD, CAT and GR activities. Only, GPx activity returned to control level in both starvation/refeeding regimes. Generally, starvation periods induced higher enzymatic activities than refeeding periods. Therefore, ROS were induced during starvation/refeeding regimes and antioxidant defense mechanisms were activated as an adaptive response. Similarly, the induction of antioxidant cell protective mechanisms is regarded as an “adaptation strategy related to the fact that many fish experience periods of starvation as part of their natural life cycle” [24]. Moreover, only GST activity was significantly reduced both by starvation and refeeding, suggesting that this antioxidant enzyme was not important in the detoxification of electrophilic compounds. Unfortunately, even though both SOD-CAT and GPx-GR antioxidant defense mechanisms were activated during starvation and the activities of several enzymes remained high after refeeding (e.g., CAT and GR), a certain amount of ROS managed to escape the enzymatic scavenging systems and affected some biomolecules. Lipids were more susceptible to ROS mediated oxidation than proteins. The lipid peroxidation did not return to control level after refeeding, while protein oxidation biomarkers almost reached the control levels or were similar to them after refeeding. Therefore, the starvation period might have been too long or the refeeding period might have been too short to allow a complete recovery of the oxidative stress biomarkers and antioxidant enzymes in the intestine of stellate sturgeon. Similar to our results, it was reported an increase of lipid peroxidation level and of all antioxidant enzymes excepting GST in the liver of starved *Salmo trutta*, and the increase trend was still observed at the end of a 21-day refeeding period. Therefore, in agreement to our results observed for the intestine of *Acipenser stellatus*, total food deprivation induced oxidative stress and the effects of food restriction did not disappear even after a 21-day refeeding period in *Salmo trutta* liver [42]. Furthermore, starvation induced oxidative stress reflected as an increase of lipid peroxidation level in the liver of *Acipenser naccarii* and *Oncorhynchus mykiss*. This modification was accompanied by a decreasing trend in the enzymatic activities of CAT, SOD, GPx and GR. The stress was not removed after subsequent refeeding, thus the liver of these two fish species showing an “incapacity to meet the stress situation provoked by 72 days of starvation, leading to the oxidation of the biomolecules” [46]. Additionally, it was reported an enhancement of the total antioxidant capacity of the blood in *Acipenser sinensis* in the first 19 days of starvation and after that period a reduction was observed [56].

In contrast to our results, lipid peroxidation level indicated that the oxidative stress disappeared and the antioxidant enzymatic activities returned to control values after refeeding thus showing a “compensatory capacity” in *Dentex dentex* [41], *Mesopotamichthys sharpeyi* [45] and *Sparus aurata* [44].

In addition, the present work extends a previous study conducted by our research group on stellate sturgeon liver regarding the adaptability of this species to a starvation/refeeding regime. Our previous data had shown that *A. stellatus* juveniles subjected to 7/21-day starvation/refeeding regime presented a complete compensatory growth and were able to efficiently counteract the oxidative stress by enhancing activities of the antioxidant enzymes in their liver [25]. Only the 7 S-R group presented a complete recovery of the weight loss during refeeding, as reflected by the final weight, weight gain and specific growth rate. All of the morphometric parameters suggested that 7 S-R group reached the same weight of the fed control and that the 7/21-day starvation/refeeding regime did not affect the growth performance of *A. stellatus* juveniles [25]. Additionally, ROS were induced only after 14 days of starvation, leading to enhanced activity of antioxidant enzymes such as: CAT, GR and GST in the liver of *A. stellatus*. However, ROS were entirely neutralized by the antioxidant enzymes and even more, since the lipid peroxidation decreased and proteins were spared from oxidation during starvation [25]. Refeeding induced ROS to a greater extent than starvation and gradually activated almost all antioxidant enzymes in the liver, so the lipid peroxidation increased, but did not surpass the control level, while protein oxidation occurred only when juveniles were refed after 14-day starvation [25]. 

In the present study, it was observed that 7/21-day starvation/refeeding regime induced oxidative stress to a smaller extent in the intestine of stellate sturgeon than 14/21-day starvation/refeeding regime did. Based on our previous data and on the fact that the antioxidant enzymatic mechanisms were enhanced and the fact that the raise of lipid peroxidation level was statistically insignificant in the intestine of the juveniles subjected to 7/21-day starvation/refeeding regime, we concluded that this alternative feeding regime is worth to be further researched and optimized. Therefore, *Acipenser stellatus* juveniles possess a potential to adapt to a short starvation period introduced in the feeding schedule. 

Moreover, *Acipenser stellatus* juveniles might be able to adapt to long-term starvation. A peculiar observation regarding our data is that the level of some oxidative stress biomarkers (SOD, GSH, MDA, PTG, PCG and AOPP) was not statistically different between the fed and starved controls. These findings were also observed and reported in our previous study conducted on stellate sturgeon liver [25]. These results could be interpreted as an adaptation of the juveniles to long-term starvation. Mature *A. stellatus* individuals possess the ability to cope with starvation in the wild. These are capable to survive months without feeding during the migration and reproductive seasons because they accumulate energy stores during feeding season [1]. Additionally, fish alternate feeding with starvation periods because of seasonal variations in temperature and food availability [10]. This observation has not been made before for juveniles that need energy resources for growth and development, and that are supposed to present a continuous feeding behavior. However, wild juveniles might experience short periods of food deprivation because of the food availability, so they might not eat on a daily basis. Therefore, *A. stellatus* juveniles might possess an innate capacity to cope with starvation for longer periods. 

Our study must be regarded as a starting point in optimizing stellate sturgeon feeding in aquaculture due to some limitations we confronted with. One limitation of our study consists in large values of standard deviation and standard error of the mean due to a high degree of variability amongst individuals. It seems that adaptive responses to starvation/refeeding regimes differ to a high degree between individuals. Unfortunately, there was no possibility to raise the number of individuals per group due to the necessity to protect the species which is on the verge of extinction. In addition, it is very expensive to raise stellate sturgeon juveniles in aquaculture and it is very hard to successfully reproduce sexually mature individuals. Due to the need to spare sacrification of large number of individuals and to reduce the number of animals used for ethical purpose we lowered the number of individuals as much as it was scientifically possible and used only one control group for all treatments. In consequence, the experiment was not repeated to validate the results. Therefore the issue of pseudo replication and the issue of using a unique control group represent the second limitation of the study. This study has to be extended if possible and it represents, as mentioned above, a starting point in optimizing the feeding practice of *Acipenser stellatus*.

One future research direction of our study consists in analyzing Hsp stress response to starvation/refeeding regimes. Additionally, it is our desire to extend the study on other organs. Furthermore, it is highly important to conduct this experiment in such a manner that it could test the ability of stellate sturgeon to adapt to long-term applied starvation/refeeding regimes. 

A starvation/refeeding regime has certain economical and scientific benefits. If applied in aquaculture, such a regime could lower the costs of raising juveniles and enhance the profitability of fish farms without harming the juveniles. In the long term, an enhanced profitability might lead to an increased number of fish farms, which could stimulate the conservation of the stellate sturgeon. Therefore, a starvation/refeeding regime represents a strategy aimed to improve the sustainability of fish farming. This alternative type of fish feeding is worth taking into consideration for both the socio-economical implications and conservation of the stellate sturgeon. 

## 5. Conclusions

In conclusion, the antioxidant defense mechanisms were enhanced by starvation/refeeding regimes in the intestine of *Acipenser stellatus*. Moreover, the 7/21-day starvation/refeeding regime induced oxidative stress to a lesser extent than the 14/21-day starvation/refeeding one did. According to our data, *Acipenser stellatus* possess a potential to adapt to a short starvation period introduced in the feeding regime. The results of the present study endorse that a starvation/refeeding regime is an alternative feeding strategy worthy of being further researched, optimized and eventually applied in aquaculture.

## Figures and Tables

**Table 1:** The biochemical composition of Troco Pre Grower commercial pellets.

Substance	Quantity
Proteins	45%
Lipids	18%
Raw fiber	1.2%
Ash	8.2%
Phosphorus	1.2%
Calcium	1.8%
Sodium	0.4%
Vitamin A	10.000 I.E
Vitamin D3	746 I.E
Vitamin C	150 mg/kg
Vitamin E	200 mg/kg

## Data Availability

Data available on request due to restrictions. The data presented in this study are available on request from the corresponding author. The data are not publicly available due to privacy reason.
